# A resource for sustainable management: *De novo* assembly and annotation of the liver transcriptome of the Atlantic chub mackerel, *Scomber colias*

**DOI:** 10.1016/j.dib.2018.03.013

**Published:** 2018-03-13

**Authors:** André M. Machado, Mónica Felício, Elza Fonseca, Rute R. da Fonseca, L. Filipe C. Castro

**Affiliations:** a CIIMAR – Interdisciplinary Centre of Marine and Environmental Research, U. Porto – University of Porto, Porto, Portugal; b Portuguese Institute for the Sea and Atmosphere, I.P. (IPMA), Portugal; c Department of Biology, Faculty of Sciences, U. Porto – University of Porto, Portugal; d The Bioinformatics Centre, Department of Biology, University of Copenhagen, Denmark

**Keywords:** RNA-Seq, Scombridae, Stock management, Atlantic chub mackerel, Liver

## Abstract

Mackerels represent a valuable fishery worldwide. Their ample geographic distribution and capture levels make them an insightful model to address stock management strategies in the context of global changes. Yet, and despite recent impressive genome and transcriptome sequencing efforts from teleost species, available resources from the Scombridae family are comparatively scarce. Here, we generated the first high-quality *de novo* assembly of the liver transcriptome of the Atlantic chub mackerel (*Scomber colias*). Through the use of RNA-Seq Illumina technology, 111,124,228 clean reads were obtained for the liver transcriptome. *De novo* assembly resulted in 93,731 transcripts with an N50 of 1462 bp. This dataset provides an important insight into the context of fisheries management.

**Specifications Table**

**Table:** 

**Subject area**	**Genetics and Transcriptomics**
**More specific subject area**	Transcriptomics of Atlantic chub mackerel Liver
**Type of data**	Raw reads of DNA sequences
**How data was acquired**	A liver sample of Atlantic chub mackerel, *Scomber colias,* was collected for total RNA isolation. It was prepared paired-end library and sequenced by the Hiseq. 4000 system. The obtained data were subjected to *de novo* assembly and annotated using Trinotate.
**Data format**	Raw data in FASTQ, transcriptome assembly and Final transcriptome assembly in FASTA format.
**Experimental factors**	One specimen of *S. colias* was obtained from North Atlantic waters.
**Experimental features**	The *de novo* assembling of the transcriptome, decontamination, filtration and the functional annotation of Atlantic chub mackerel liver was performed.
**Data source location**	Portugal (41.501944 N 8.851667 W)
**Data accessibility**	The raw FASTQ files were deposited in the NCBI SRA database with accession number SRX3462868 (https://www.ncbi.nlm.nih.gov/sra/SRX3462868).
	The decontaminated transcriptome assembly was deposited in the NCBI TSA database with accession number GGCI00000000.1 (https://www.ncbi.nlm.nih.gov/nuccore/GGCI00000000.1).
	The final transcriptome assembly was deposited in the figshare digital repository (https://figshare.com/s/a97f1d5b37d174d1d819).

**Value of the data**

• This is the first high-quality *de novo* assembly of the liver transcriptome of the Atlantic chub mackerel.
• The transcriptome results presented here pave the way for developing the appropriate tools fundamental for resource management of *S. colias*.
• The on-going full genome sequence project will largely profit from this dataset at the annotation stage.

## 1 Data

Teleostei, an infraclass of the Actinopterygii, comprise by far the most species-rich group within the vertebrates, with more than 26,000 recognized species [1]. Their fantastic variation in morphology and physiology traits is paralleled by the plethora of colonized aquatic habitats. Moreover, teleost species are a critical component of human diets providing nutrients such as proteins and lipids, including the essential “omega 3” [2], [3]. Recently, a massive effort in the gathering of full genome sequences from 66 teleost species has been performed [2], [3]. In addition, the implementation of international collaborative initiatives with the aim to generate large-scale and comparable datasets of RNA-Seq transcriptome sequences, such as the “*Transcriptomes of 1000 Fishes”* (Fish-T1K; https://db.cngb.org/fisht1k/home/), is also noteworthy [4]. Mackerels from the genus *Scomber* comprise a substantial proportion of the total volume of captured fish worldwide [5], since they are highly appreciated by consumers. In Portugal, the Atlantic chub mackerel, *Scomber colias*, represents the species with the highest volume of capture recorded in 2016 (>26,000 t) [6]. Thus, the transcriptomic dataset presented here provides important information for comparative genomics across the teleost tree of life, the definition of stock management strategies, and to investigate the biology and ecology of this important economic species. The provided dataset consists of raw reads of Atlantic chub mackerel*,* deposited in NCBI SRA database under SRX3462868 accession number. The raw reads were *de novo* assembled into full-length transcriptome, that after filtration, decontamination and quality control were deposited in NCBI TSA database with GGCI00000000.1 accession number. In addition, we produced and annotated a filtered transcriptome assembly, final Transcriptome Assembly, derived from the previous one. Importantly, all steps of data treatment were supported by statistical analyses showed in several tables and figures.

## 2 Experimental design, materials and methods

### 2.1 Atlantic chub mackerel collection, sampling, and Illumina sequencing

One specimen of *S. colias* was obtained from North Atlantic waters during the “Programa Nacional de Amostragem Biológica” carried out by the Instituto Português do Mar e da Atmosfera” (IPMA) (Table 1). The liver tissue was sampled immediately upon capture, stored in RNAlater, and kept at −20 °C until RNA extraction. Total RNA (RNAt) of the liver was extracted using the RNeasy Mini Kit (Qiagen) with a pre-treatment with DNaseI and subsequent elution of the extracted RNAt in nuclease-free water, according to the manufacturer's protocol. A strand-specific library with an insert size of 250–300 bp was built after conversion of the high-quality liver RNAt to cDNA and sequenced using 150 bp paired-end reads on the Illumina HiSeq. 4000 platform by Novogene (China).

**Table 1:** Transrate and Trinity Statistics of the original, decontaminated and final transcriptome assembly of liver transcriptome of *S. colias*.

**Trimming & Assembly**	**Liver tissue**		
**Raw sequencing reads**	121,656,039		
**Reads used in assembly**	111,124,228		
**Percentage of reads submitted to assembly**	91.34%		
**Assembly Versions**	**Original transcriptome assembly**	**Decontaminated transcriptome assembly**	**Final transcriptome assembly**
**Number of “genes”**	72,618	54,876	35,386
**Number of transcripts**	114,174	93,731	44,345
**n50 transcript length (bp)**	1299	1462	1288
**Median transcript length (bp)**	544	593	593
**Mean transcript length (bp)**	899	975	912
**Smallest Contig**	301	301	301
**Largest Contig**	14,405	14,405	13,899
**Number of Contigs over 1k nn**	30,346	28,819	12,962
**Number of Contigs over 10k nn**	28	26	3
**GC %**	45.33	45.32	46.64
**Total Assembled bases**	102,639,256	91,425,968	40,443,374
**RMBT %**	–	–	81.37%

### 2.2 Transcriptome data processing and de novo assembly

The raw reads of liver tissue were produced by sequencing and quality filtered by Trimmomatic [7], with parameters set to “LEADING:15 TRAILING:15 SLIDING WINDOW:4:20 MINLEN:50”. The statistics of trimming reads are shown in Table 1.

Since the reference genome of *S. colias* is not currently available, the Trinity v2.4.0 software was used for *de novo* assembly [8]. We applied the software following the protocol from Hass and colleagues [9], with exception of the strand-specific data and minimum length contig parameters (SS_lib_type RF; min_contig_length 300).

To check the raw assembled contigs for contamination, the assembled transcriptome was queried in the MCSC decontamination pipeline [10] with the following parameters: LVL =5; TAXO_LVL=superclass; WHITE_NAME= Actinopterygii, and all contigs with a match to Actinopterygii sequences of Uniref90 database were kept. The remaining contigs with a match to other taxa or without hits at all, were re-blasted against the nucleotide database (NT) of NCBI, with an E-value cut-off of 1e-5. Again, contigs having top hits outside of Actinopterygii taxa were excluded, while the contigs without hits at all were retained. To check and remove vector sequences, adapter and linkers, not previously identified, we also filtered the assembled transcripts against the UniVec database. Any sequences of our assembly with a strong match against UniVec database (1/1,000,000 chance of a random match for queries of 350 Kb, terminal match score ≥24, internal match score ≥30) were removed.

**Fig. 1:**
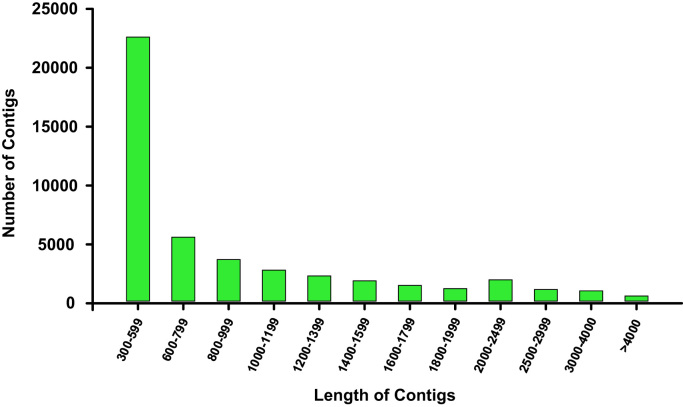
Length distribution of the final transcriptome contigs. The x-axis represents the length, and the y-axis represents the number of Contigs.

To decrease the isoform redundancy of the clean assembly, the tr2aacds pipeline, from the Evidential – Gene package (http://arthropods.eugenes.org/EvidentialGene/), was used. This pipeline reduces redundancy by selecting the ‘optimal’ set of assembled transcripts, based on coding potential. For each filtration stage of the assembly, the Trinity and Transrate [11] statistics are shown in the Table 1. Furthermore, and despite the redundancy removal and decontamination clean-up steps in the initial stages, the rate of reads mapped back to transcripts (RMBT) as well the distribution length of final assembled sequences was calculated and plotted as a measure of assembly quality (Table 1; Fig. 1).

To assess the completeness of our transcriptome, in terms of gene content, the Benchmarking Universal Single-Copy Orthologs tool (BUSCO) was used [12].The statistics of complete BUSCO hits against the eukaryota and metazoa lineage-specific profile libraries are provided in Table 2.

**Table 2:** BUSCO statistics of completeness of S. colias Liver final transcriptome assembly against the metazoa and eukaryota gene sets.

**BUSCO Statistics**	**Metazoa DB (%)**	**Eukaryota DB (%)**
**Complete**	82.90	83.20
**Single**	73.60	71.60
**Multi**	9.30	11.60
**Fragment**	13.00	13.20
**Missing**	4.10	3.60

### 2.3 Functional annotation

**Fig. 2:**
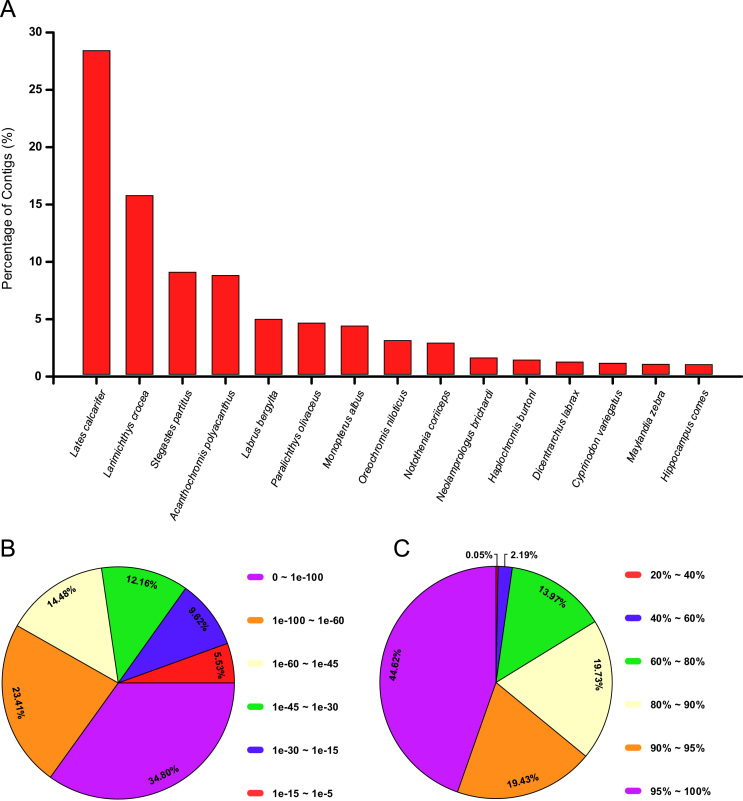
Blastx analysis of *Scomber colias* final transcriptome assembly. (A) Homologous gene-species distribution (B) E-value distribution, (C) Similarity distribution.

The functional annotation was performed in two independent steps. Firstly, the final transcriptome assembly was queried against the non-redundant (NR) database of NCBI, through the blastx tool of DIAMOND v0.8.36 software [13] and applying an E-value cut-off of 1e-5. The top 30 species with the blastx best hits are provided in Table 3. To facilitate the visualization, only the top 15 best blast hits are plotted in the species distribution in Fig. 2A. Moreover, and to strengthen the blast analysis, the E-value and sequence similarity distributions were also tabulated and displayed in Fig. 2B and C and in Table 4, Table 5.

**Table 3:** The top 30 species for which there was a top blastx hit. These blastx results from the queried of final transcriptome assembly against Non-Redundant Database of NCBI.

**Specie**	**Taxon ID**	**Number of blastx Hits**	**Percentage of blastx Hits (%)**
***Lates calcarifer***	8187	8,503	28.20
***Larimichthys crocea***	215358	4,696	15.58
***Stegastes partitus***	144197	2,683	8.90
***Acanthochromis polyacanthus***	80966	2,597	8.61
***Labrus bergylta***	56723	1,447	4.80
***Paralichthys olivaceus***	8255	1,346	4.46
***Monopterus albus***	43700	1,270	4.21
***Oreochromis niloticus***	8128	822	2.73
***Notothenia coriiceps***	8208	551	1.83
***Neolamprologus brichardi***	32507	431	1.43
***Haplochromis burtoni***	8153	375	1.24
***Dicentrarchus labrax***	13489	322	1.07
***Cyprinodon variegatus***	28743	290	0.96
***Maylandia zebra***	106582	262	0.87
***Hippocampus comes***	109280	256	0.85
***Fundulus heteroclitus***	8078	255	0.85
***Cynoglossus semilaevis***	244447	220	0.73
***Kryptolebias marmoratus***	37003	205	0.68
***Austrofundulus limnaeus***	52670	203	0.67
***Oryzias latipes***	8090	199	0.66
***Nothobranchius furzeri***	105023	175	0.58
***Poecilia latipinna***	48699	164	0.54
***Boleophthalmus pectinirostris***	150288	152	0.50
***Tetraodon nigroviridis***	99883	151	0.50
***Pundamilia nyererei***	303518	144	0.48
***Oncorhynchus mykiss***	8022	142	0.47
***Poecilia mexicana***	48701	142	0.47
***Takifugu rubripes***	31033	139	0.46
***Cyprinus carpio***	7962	133	0.44
***Oplegnathus fasciatus***	163134	120	0.40

**Table 4:** E-value distribution of blastx hits of final transcriptome assembly against NR database.

**E-values Ranges**	**Number of blastx Hits**	**Percentage of blastx Hits (%)**
**0 ~ 1e-100**	10492	34.80
**1e-100 ~ 1e-60**	7057	23.41
**1e-60 ~ 1e-45**	4366	14.48
**1e-45 ~ 1e-30**	3667	12.16
**1e-30 ~ 1e-15**	2899	9.62
**1e-15 ~ 1e-5**	1667	5.53

**Table 5:** Similarity distribution of blastx hits of final transcriptome assembly against NR database.

**Similarity Ranges (%)**	**Number of blastx Hits**	**Percentage of blastx Hits (%)**
**20 ~ 40**	15	0.05
**40 ~ 60**	660	2.19
**60 ~ 80**	4212	13.97
**80 ~ 90**	5949	19.73
**90 ~ 95**	5859	19.43
**95 ~ 100**	13453	44.62

**Fig. 3:**
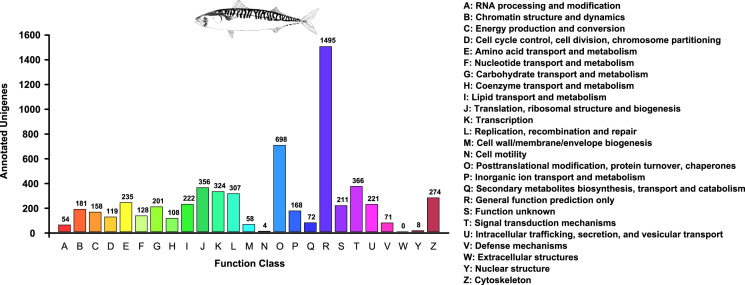
Histogram of the clusters of orthologous groups (COG).

In the second step, the nucleotide sequences of the final transcriptome were submitted to the Trinotate v3.0.1 (http://trinotate.github.io). Into Trinotate pipeline were used several annotation software such as Transdecoder (http://transdecoder.github.io), Hmmer v.3.1b1 [14], PFAM [15], TMHMM v.2.0c [16], GOseq [17] and eggNOG v.3.0 [18] to perform the functional annotation of transcriptome. Open reading frames (ORFs) were predicted using the Transdecoder. Obtained ORFs were blasted using the blastp tool of DIAMOND v0.8.36 software [13] against the NCBI NR, Uniref90, and SwissProt databases with an E-value cut-off of 1e-5. To avoid statistical gene overrepresentation, when more than one isoform per ‘gene’ remained after filtering with the tr2aacds pipeline, only the top representative of each ‘gene’ was selected for further analysis. To belong to the final subset of unigenes, a sequence would have to obey the following criteria (in this order): 1) codify an ORF, 2) display a blastx or blastp hit in at least one of the 3 chosen databases (NR, Swissprot, Uniref90), 3) represent the longest ORF per ‘gene’. The annotation statistics of final transcriptome assembly and final transcriptome subset can be consulted in Table 6. Additionally, the Clusters of Orthologous Groups (COG) screening was performed using the eggNOG database, integrated within the Trinotate pipeline. The COG distribution is available in Fig. 3.

**Table 6:** Functional annotation categories and statistics for final transcriptome assembly and for a subset of unigenes.

**Trinotate Annotation Statistics**	**Final transcriptome assembly**	**Final Transcriptome Subset**
**Number of “genes” with ORF**	21,981	–
**Number of “Unigenes” with ORF**	–	21,981
**Number of transcripts with ORF**	27,772	21,981
**Transcripts with blastx match NR**	27,426	21,707
**Transcripts with blastp match NR**	26,780	21,215
**Transcripts with blastx match Uniref90**	27,519	21,791
**Transcripts with blastp match Uniref90**	26,889	21,307
**Transcripts with blastx match SwissProt**	23,927	18,867
**Transcripts with blastp match SwissProt**	24,086	19,013
**Transcripts with GO terms**	23,550	18,567
**Transcripts with KeggPathways**	21,247	16,911
**Transcripts with eggNOG/COG**	21,005	16,754
**Transcripts with PFAM**	18,885	14,557
