# Supplementary material for: A resource for sustainable management: De novo assembly and annotation of the liver transcriptome of the Atlantic chub mackerel, Scomber colias
Source: Data Brief. 2018 Mar 13;18:276–84. doi: 10.1016/j.dib.2018.03.013 (PMC5996228; doi:10.1016/j.dib.2018.03.013)
Supplement: Supplementary file 1 — Supplementary material [file mmc1.docx]

**Conflict of Interest**

The authors declare there is no conflict of interest on any work in this paper.
